# Enhancement of Biosensors by Implementing Photoelectrochemical Processes

**DOI:** 10.3390/s20113281

**Published:** 2020-06-09

**Authors:** Melisa del Barrio, Gabriel Luna-López, Marcos Pita

**Affiliations:** 1 Departamento de Biocatálisis, Instituto de Catálisis y Petroleoquímica, CSIC. C/Marie Curie, 2. L10. 28049 Madrid, Spain; melisa.delbarrio@uam.es (M.d.B.); gabriel.luna@csic.es (G.L.-L.); 2 Departamento de Química Analítica y Análisis Instrumental, Universidad Autónoma de Madrid, Campus de Cantoblanco, 28049 Madrid, Spain

**Keywords:** biosensors, bioelectrochemistry, photo-biosensors, enzyme, biocatalysis

## Abstract

Research on biosensors is growing in relevance, taking benefit from groundbreaking knowledge that allows for new biosensing strategies. Electrochemical biosensors can benefit from research on semiconducting materials for energy applications. This research seeks the optimization of the semiconductor-electrode interfaces including light-harvesting materials, among other improvements. Once that knowledge is acquired, it can be implemented with biological recognition elements, which are able to transfer a chemical signal to the photoelectrochemical system, yielding photo-biosensors. This has been a matter of research as it allows both a superior suppression of background electrochemical signals and the switching ON and OFF upon illumination. Effective electrode-semiconductor interfaces and their coupling with biorecognition units are reviewed in this work.

## 1. Introduction

Humankind is on a permanent quest for better ways to extract relevant information from the environment. Many devices, known as sensors, have been created, designed and perfected since the ancient days aiming at increasing the knowledge and therefore allowing better decisions. Therefore, sensors have been implemented in most facets of life and have involved an incommensurable panoply of processes and systems to provide meaningful information. 

Regardless of their implementation area, some common features are desired to define efficient sensors: easiness to use, reliability and fast response are the most desired characteristics. The technological revolution accomplished since the late 20th Century has had a huge impact in the sensing sector. Sensors have improved thanks to the evolution of optical and electronic transducers. Sensors have implemented new catalytic processes and used more reliable materials to achieve a faster response, among other improved properties [1]. Biosensors are a particular case worth research, in which a chemical reaction catalyzed by a biological entity, mainly an enzyme, triggers the process to inform about the presence and concentration of a specific molecule. Enzymes provide great features to biosensors, such as selectivity and specificity, which help to ease the biosensing process by avoiding purification steps or matrix effects. Among all kinds of enzymes, redox ones are particularly well suited to being linked with electrochemical methods, as electroactive surfaces may transfer electrons from and to the enzymes; this transfer substitutes that of one of the substrates and directly correlates the enzymatic activity to electrochemical signals. This combination allows for easy, affordable and reliable processes for sensing specific analytes. Some examples of amperometric biosensors have been developed to sense key biomolecules such as adenosine triphosphate (ATP) [2] or general substrates like oxygen [3]. Enzymatic reactions can also be combined with each other to perform simultaneous analysis of biochemicals, emulating logic operations [4,5].

Classically there have been three generations of biosensors [6]. In the first generation, the enzymatic reaction takes place and the product is directly measured with the electrode. The second generation substitutes the enzyme’s substrate that is not sensed by a mediator, which accomplishes the electron transfer to the electrode and adds a catalytic effect able to increase the signal. The third generation can be achieved with enzymes, the active site of which is available for direct electron transfer. The immobilization of these enzymes on the electrode surface allows a direct electrochemical measurement of the substrate. Regardless of the biosensor architecture, there are common challenges to overcome. Selectivity is a major issue for efficient sensors to avoid false positives, which is provided by the enzymes in the case of biosensors. Another one is the sensitivity, which is related to the noise level measured in the absence of substrate. There have been many attempts to study the noise level in bioelectrochemical systems. The inclusion of a single enzyme system [7,8] or cascade-concatenated biochemical reactions [9,10] and use of strategies like the incorporation of chemical filters to suppress or delay the background signal [11] are some examples of these efforts. A way to reduce the noise level is to include a semiconductor between the electrode and the biosensing structures [12]. Moreover, semiconductors often can harvest light energy and become an electric conductor upon illumination with visible light, which has even been used for water splitting [13,14,15]. Such building allows a huge noise reduction while adding a switch system to the biosensor, improving the device performance, and yielding photo-biosensors as a new tool for better sensors [16]. Early reports of photobioelectrochemical (PEC) sensors were published more than 20 years ago [17], but it has been in more recent years when the field has blossomed into many systems for different applications. In this review we will focus on photo-biosensors for relevant analytes such as glucose, lactate, protein kinase or Acetyl Choline Esterase (AChE), among other examples.

## 2. Semiconductors Used in Photo-Biosensors

The main characteristic of photo-biosensors with respect to amperometric biosensors is the addition of a semiconducting material acting as a switch upon illumination, which can be triggered or modulated when the analyte is present. Key aspects to develop such devices are how to combine the electroactive surface, the semiconductor and the sensing biocatalysts; another key aspect is which materials are more suitable, depending on the application desired. Conventional bioelectrochemistry requires that the redox biocatalysts are connected to the electrode in some fashion. Most enzymes have the catalytic site buried inside the protein structure, so the use of mediators for transferring the electrons is a common strategy. However, there are enzymes that can be oriented on the electrode surface and achieve direct electron transfer. Photo-assisted electrochemistry adds higher complexity to the electron transfer, because semiconductors are generally not as reversible as conductors. Their p- or n-type semiconducting behavior marks the main flux of electrons, so depending on the reaction biocatalyzed only one of the semiconductor types will offer successful photo-biosensor constructions. This characteristic must be considered when selecting the semiconductors.

A very early approach utilized fused-silica optical fibers covered with gold [17]. This sensor was designed to detect catalase activity while providing its substrate, H_2_O_2_, generated from existing O_2_ upon illumination. Other successful interfaces have included quantum dots (QDs) on the surface of electrodes. Some of them use gold as electrode and load it with CdS to detect formaldehyde [18], although gold has not been the most common electrode material used for these purposes. Another alternative is to use conductive liquid contacts as electroactive material, such as a eutectic mixture of gallium and indium, which is liquid at room temperature. This has been used to contact silicon wafers etched with HF [19].

One of the most successful electrodes that has been used in many works is indium-doped tin oxide (ITO). Because tin oxide is itself a semiconductor, when doped with other ions like indium (III) or fluoride it yields a conductive surface with high transparency. These have become the most common electroactive materials for photoelectrodes, which are the bases of photo-biosensors. On the top of ITO many semiconductors have been tested, such as BiOI nanoflakes [20]; layers of graphene, chitosan-Cd^2+^ [21]; Bi_2_S_3_ [22]; CdS QDs [23]; ZnS nanoparticles [24]; TiO_2_ covered with QDs made of CdSeTe@CdS@ZnS [25]; layers of NiO and CuInS_2_ [26]; WS_2_ and gold nanoparticles [27]; laser-induced TiO_2_-decorated graphene (LITG) [28]; carbon nitride with gold nanoparticles [29]; or NiWO_4_ nanostructures [30]. It would be worth comparing these materials’ performance with the same biosensing system, but to date they have been tested for different applications, so such study is beyond today’s reported knowledge.

Many electroactive surfaces have been developed besides ITO-based electrodes, such as silicon (111) working as substrate for InGaN/GaN nanowire growth [31], which served to detect reduced nicotinamide adenine dinucleotide (NADH). Graphene combined with TiO_2_ nanowires has also been used as substrate on the top of polymer nanosponges, becoming conductive and photoactive. Another strategy is an oxidizing etching-annealing of a metal foil’s surface, which has been applied to copper, yielding a CuO nanotubes’ coverage [32], and titania, forming a TiO_2_ cover [33]; the latter has been also used in combination with chitosan to favor the cross-linking of enzymes like horseradish peroxidase (HRP) [34]. Photoactive polymers like polythiophene derivatives have also been tested [35] combined with CdS QDs. Another example of photoelectrode for biosensing has been demonstrated using a Ni:FeOOH/BiVO_4_ photoanode [36].

Overall, the combination of conductive materials with photoactive materials is a key aspect to look at when developing photo-biosensors. There are many options to suit specific needs, and it is still a field that remains open to new composites to be developed and/or discovered.

## 3. Enzymes Immobilization and Performance

Enzymes play a major role in any kind of biosensors, as they are the recognition unit responsible to provide the information to the transducing system. The enzymatic reaction should take place close enough to the photoelectrode surface to transfer the chemical information either via direct electron transfer or by means of a mediator, so its immobilization in the surface or close to it is a very common strategy. Many enzymes have been used for different sensors. Very common analytical targets are glucose, lactate, kinase-like proteins or acetylcholine esterases, although they are not the only ones.

### 3.1. Glucose Detection

Glucose biosensors have become important devices in the medical field due to their contribution against diabetes mellitus. Many different strategies have been developed to address this problem. In the following section, some of the newer approaches and strategies will be reviewed. Furthermore, the performance of the resulting photo-biosensors is summarized in Table 1.

Ren et al. used ZnO nanoparticles bound to glucose oxidase (GOx) due to its biocompatibility, photoconductivity, photocatalytic activity and high electron transfer capacity [37]. These ZnO nanoparticles were multigrain and the hexagonal phase of ZnO suited best for GOx immobilization, probably due to its larger surface to volume ratio. The adsorption process did not significantly affect the secondary structure of the macrobiomolecule. The effects of ZnO nanoparticles were monitored by amperometric measurements. A control experiment lacking ZnO nanoparticles yielded a current response of 0.82 µAcm^−2^, while ZnO presence provided up to 21 µAcm^−2^. The larger surface of the ZnO nanoparticles intensely enhances the current activity of the electrode through a better adsorption of GOx. The optimal conditions for these biosensors were pH 6.8 and 45 °C, although the system was also successful at 35 °C, which allows compatibility with the human body. They also discovered that the current density increased upon irradiating UV light to the sample, reaching 27 µAcm^−2^. Further experiments showed that the current increased up to 30% more when irradiated, but the photo-bioelectrode inactivated after a long exposure to light, probably due to denaturation of the enzyme.

**Figure 1:**
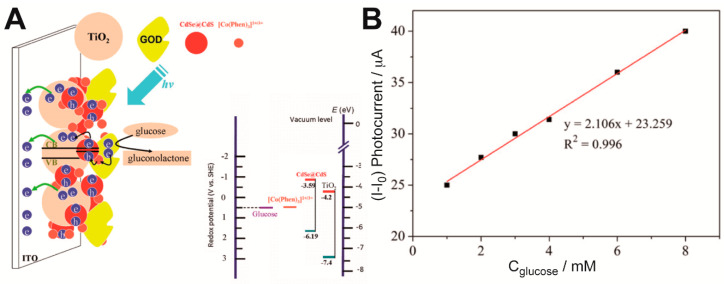
(**A**) Schematic of the photo-bioelectrode building based on QDs and GOx, and the energy levels of its components. (**B**) Linear response of the sensor. Reprinted with permission from ref. [39].

Sun et al. manufactured a new photoelectrochemical biosensor based on CdS nanoparticles [38]. Polyamidoamine (PAMAM) dendrimer was used as inner template to synthesize CdS nanoparticles. GOx was immobilized on Pt electrodes together with the CdS nanoparticles through layer-by-layer (LbL) technique. This immobilization method consisted of adsorbing sequentially charged macromolecules, where PAMAM acted as scaffold for the CdS nanoparticles to grow. Along with GOx it formed the glucose-detecting electrode in aqueous solution. Platinum nanoparticles were used as charge separator. Nafion was used both as ion exchange matrix and as interference barrier. The electrodes’ performance improved under UV light irradiation at 350 nm. They have achieved twice the current response under UV light compared to dark tests. The stability of the sensor, checked every 2 days for a month, showed that the GOx immobilization on the electrode was highly effective. Another photoelectrochemical biosensor was based on a TiO_2_CdSe@CdS QDs nanocomposite electrode (Figure 1) [39]. Despite using a different kind of quantum dots, glucose was detected with GOx and the biosensor was assembled through a LbL process. The junction of TiO_2_ with CdS QDs improved the charge separation, and therefore increased the photocurrent. Furthermore, the addition of electron mediators such as [Co(Phen)_3_]^2+^ enhanced the photocurrent by suppressing the electron-hole recombination process. The sensors built this way showed a linear range from 1 to 8 nM of glucose and the lowest concentration of glucose detected was 0.05 mM, although that range and limit of detection could be improved through the optimization of the number of [Co(Phen)_3_]^2+^ and GOx bilayers. Regarding stability, this sensor was able to retain 95% of its activity after 3 weeks of storage at −20 °C.

Wang et al. developed a photoelectrochemical biosensor built around CdTe QDs capable of detecting glucose [40]. The CdTe QDs are settled on a fluorine-doped tin oxide (FTO) electrode. Then GOx is covalently attached to CdTe via amide reaction, making it able to work under visible light. This sensor showed a broad linear range and a high upper detection limit. The QDs exhibited an emission peak at 650 nm and an important absorbance shoulder at 627 nm. The experimental conditions set for this sensor were −0.2V vs. SCE, pH 7, and room temperature. The wavelength of the incident light was set to 505 nm. The accuracy and detection limits of the sensor make it promising for measuring glucose in blood in the future.

Another glucose biosensor was developed combining GOx, ITO and ZnS nanoparticles [41]. The main goal was to be able to replace CdS as the main semiconductor in the sensor due to its toxicity and potential harm to the environment. On the surface of the ITO electrode, ZnS nanoparticles were electrodeposited. Then the enzyme was immobilized on the electrode via sol-gel method. The authors proved that the immobilized GOx maintained its tertiary structure and catalytic activity. Photoelectrochemical activity from ZnS nanoparticles resulted in an improved sensibility and lower detection limit when irradiated as compared to experiments without any source of light. In addition, the sensor proved to be stable and retained its activity throughout time. 

A photoelectrochemical biosensor based on TiO_2_ nanowires and GOx was developed by Tang et al. [42]. Their single-crystalline rutile-phased TiO_2_ was hydrothermally grown on an FTO electrode. Then GOx was attached to its surface through silane/glutaraldehyde linkage. Experiments carried out with commercial TiO_2_ nanoparticles revealed that the sensitivity obtained with TiO_2_ nanowires-GOx sensors was clearly higher. Therefore, the sensitivity enhancement was directly related to the TiO_2_ nanostructure. They also evaluated the effect of interference molecules (metal ions, amino acids, glucose analogues, etc.) and their influence proved to be minimal. Finally, they tested the biosensor performance with mice serum, with remarkable results. 

A novel photobiosensor built with ZnO inverse opal photonic crystals (IOPCs) was developed by Xia et al. [43]. ZnO IOPCs have a uniform porous distribution and a massive surface area due to their structure. These crystals were obtained via sol-gel method using polymethylmethacrylate (PMMA) as a building scaffold and an FTO electrode. Then Nafion and GOx were attached to the surface and the lining of ZnO. This type of biosensor harnesses the “slow light effect” and multiple scattering from ZnO IOPCs to increase light absorption. This sensor layout proved to be highly selective, sensitive and reproducible. 

Dilgin et al. developed a glucose biosensor composed of electropolymerized hematoxylin (p-HT) film on PAMAM dendrimers that were adsorbed on a glassy carbon electrode (GCE) [44]. Then glucose dehydrogenase (GDH) was immobilized onto the whole ensemble. The electrode depends on the electrocatalytic oxidation reaction of NADH. They used a halogen lamp and flow injection analysis (FIA) to carry out the experiments. The main advantages of this technique are: low sample consumption, fast analysis and suitability for the analysis of species that would involve arduous operations of separation and chemical conversions. This sensor electrode is remarkably sensitive, selective and durable, and has proven limit of detection enhancement under irradiation.

Ertek et al. proposed photo-biosensors based on GDH and electrodeposited ZnS-CdS QDs on both multiwalled carbon nanotube modified GCE [45] and pencil graphite electrodes (PGE) [46]. Cyclic voltammetry and FIA were employed to assess the performance of the biosensors under visible radiation generated by a 250 W halogen lamp. The GCE-based photo-biosensor showed a narrower linear range (from 0.010 to 2.0 mM of glucose) compared to that of PGE-based system (from 0.2 to 8.0 mM of glucose), but a lower detection limit (4.0 µM); the latter electrode offered a higher detection limit (0.05 mM). These results suggest that these two biosensors could work complementarily.

One of the main advantages of self-powered biosensor is that there is no need to apply any voltage to the cathode and the anode. Dai et al. [47] designed a self-powered cathodic photo-biosensor focused on a hybrid PbS QDs/nanoporous NiO film nanostructure. They used ITO electrodes upon which they hydrothermally built 3D NiO nanostructures and then attached thioglycolic acid (TGA) capped PbS QDs to form a p-type heterostructure. P-type semiconductors are less prone to react with reductive interference substances. Finally, GOx was immobilized on the electrode via succinimide coupling reaction between NH_2_ groups in the enzyme and COOH groups on the surface of TGA-capped PbS QDs. The resulting biosensor proved to be highly selective, stable and sensitive and provided a fast response.

Liu et al. synthesized a composite comprising g-C_3_N_4_ and TiO_2_ bidimensional nanosheets [48]. Each of these components compensates the flaws of the other. g-C_3_N_4_ enhances the mediocre visible light excitation of TiO_2_ and the latter delays the otherwise rapid charge recombination from g-C_3_N_4_. They constructed a biosensor to evaluate the performance of this new composite in combination with ITO electrodes and GOx. Nafion was used as a binding agent to secure the enzyme to the electrode. The photoelectrochemical efficiency rose to 350% when compared to the g-C_3_N_4_ or TiO_2_ alone.

**Figure 2:**
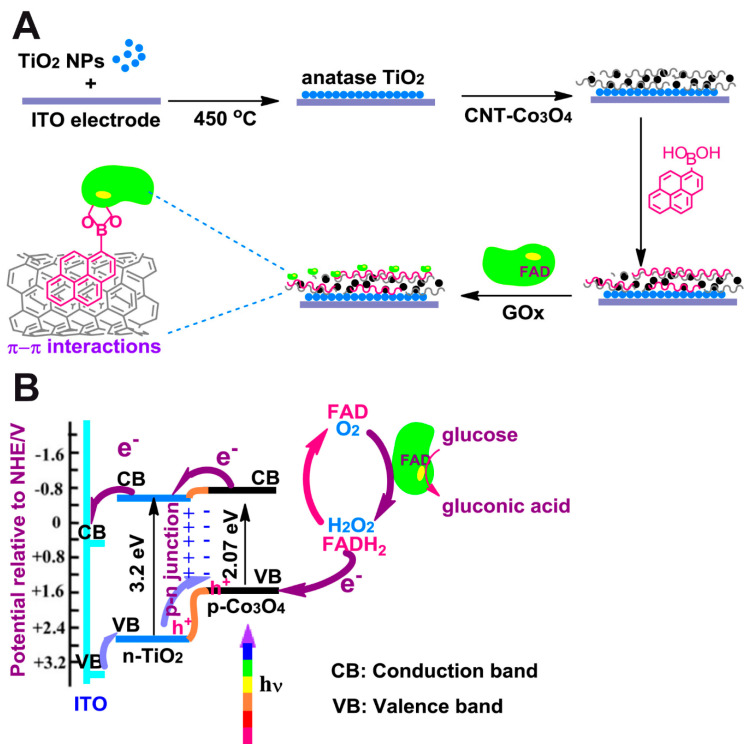
(**A**) Schematics of the photo-biosensor synthesized step by step. (**B**) Energy transfer diagram for the work showing the valence bands and conduction bands of the photo-biosensor components. Taken with permission from ref. [49].

Çakıroğlu et al. developed a self-powered biosensor [49] (Figure 2). Co_3_O_4_ and carbon nanotubes (CNT) were deposited on an ITO electrode coated with TiO_2_ anatase, creating a p-n junction. Then CNTs were functionalized with 1-pyrenic boronic acid so that a covalent bound between this moiety and the carbohydrate groups of GOx could take place through an esterification reaction. Since it is a self-powered biosensor, no external potential is needed. While normally TiO_2_ would need UV light for electrons to overcome its wide band gap, Co_3_O_4_ enhanced its photoelectrical capabilities under visible light. A linear range from 0.2 µM to 4 mM glucose concentration and a limit of detection of 0.20 µM were reported at 0 V.

Ryu et al. proposed a photoelectrochemical platform based on hematite (⍺-Fe_2_O_3_), a visible light active catalyst which was immobilized upon a FTO electrode through a two-step annealing process [50]. Polydopamine (PDA) was used to immobilize the redox mediator Nile Blue (NB) for the electrocatalytic NADH oxidation. The resulting platform was suitable for biosensing of glucose, ethanol or lactate by selecting the appropriate enzyme. GDH was used as biocatalyst for the sensor. The photo-biosensor showed a reasonable limit of detection and sensitivity, great selectivity and promising future applications.

In another example, GOx was successfully entrapped in Nafion and deposited a on a rutile nanorod/anatase nanowire TiO_2_/FTO photoelectrode [51]. The use of these TiO_2_ phases (rutile and anatase) improved the PEC glucose biosensor performance as this structure facilitated the loading of the enzyme and favored the transport of electrons from the conduction band on anatase TiO_2_ to that of rutile TiO_2_. Glucose could be detected in a relatively broad concentration range (1–20 mM) and with low limit of detection (0.019 mM).

Atchudan et al. developed an ultrasensitive PEC biosensor that comprised a novel nanocomposite of nitrogen-doped carbon sheets (NDC) wrapped titanium dioxide nanoparticles (NDC-TiO_2_ NPs) and GOx covalently immobilized on it [52]. Interestingly, the NDC-TiO NPs were synthetized from peach extract by a new green method. The energy levels of both the valence and conduction bands of NDC are at a higher level than those of TiO_2_ NPs, which favored the migration of generated electrons and holes and minimized their recombination. Regarding the PEC sensing mechanism, the H_2_O_2_ molecules—formed on the photo-bioelectrode surface from O_2_ during the GOx-catalyzed oxidation of the analyte—acted as electron donors and those electrons were transferred to the ITO electrode, while the photogenerated holes migrated from the valence band of TiO_2_ NPs to that of NDC. The H_2_O_2_ oxidation photocurrent increased linearly upon addition of glucose in the range from 50 nM to 10 μM. The PEC biosensor showed excellent selectivity, reproducibility, stability, and durability. The detection limit was as low as 13 nM. Moreover, the biosensor was capable of analyzing glucose levels in real human serum.

A nanocomposite of porous carbon nitride modified with cobalt phosphide nanoparticles (CoP/PCN) was as well proposed as photo-electroactive material and support for GOx [53]. The CoP were employed to increase the PEC response upon visible light—given that it is a good photosensitizer—and also served as electron acceptors to accelerate charge separation. In their approach, the decrease in the concentration of dissolved O_2_, which is consumed during the enzymatic reaction, and the subsequent loss of O_2_ reduction photocurrent were used for the determination of glucose. Their PEC biosensor showed a linear response in the range from 0.05 to 0.7 mM and a detection limit of 1.1 μM.

Zhang et al. proposed a bi-enzymatic glucose sensor based on graphitic carbon nitride and ZnIn_2_S_4_ composites (g-C_3_N_4_/ZnIn_2_S_4_) and a biocatalyzed precipitation reaction [54]. GOx and horseradish peroxidase (HRP) were immobilized, with the aid of gold nanoparticles, on a glassy carbon/g-C_3_N_4_/ZnIn_2_S_4_ photoelectrode, in which GOx catalyzed the oxidation of glucose to generate H_2_O_2_. In the presence of 4-chloro-1-naphthol (4-CN), HRP used H_2_O_2_ to catalyze the oxidation of 4-CN to form an insoluble compound (benzo-4-chlorohexadienone). The formed precipitate acted as a barrier towards electron transfer between g-C_3_N_4_/ZnIn_2_S_4_ and an electron donor (L-cysteine, which trapped the photogenerated holes of the semiconductors), accelerated the carrier recombination and, as a consequence, the oxidation photocurrent of the electron donor decreased. They found a linear relationship between the photocurrent and the logarithm of glucose concentration in the range 1–10,000 μM and a low detection limit of 0.28 μM. This novel methodology was also applied for the determination of glucose in diluted human serum.

Furthermore, a study by Chen et al. concerning the photocurrent switching effect of BiVO_4_ semiconductors (i.e., the p-type semiconductor behavior of this typical n-type semiconductor at a bias potential) led to the design of a PEC glucose sensor [55]. The sensing strategy was based on the measurement of the reduction photocurrent of H_2_O_2_ produced during the GOx-catalyzed glucose oxidation. Their FTO/BiVO_4_/GOx photoelectrode exhibited excellent selectivity, high sensitivity (the detection limit was 0.73 μM) and was barely affected by oxygen level fluctuations.

**Figure 3:**
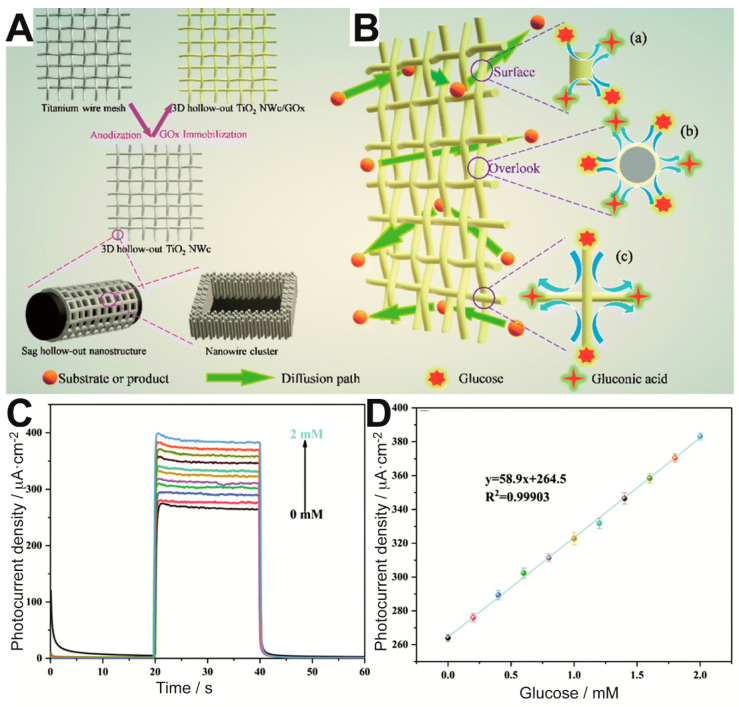
Schematic of (**A**) the preparation of 3D hollow-out TiO_2_ NWc/GOx electrode and (**B**) glucose detection on the mesh electrode. Insets (a–c) show that the 3D network structure allows GOx to perform catalysis on a high surface. (**C**) Photocurrent density response over time of 3D hollow-out TiO_2_ NWc/GOx in the presence of increasing concentrations of glucose. (**D**) Calibration plot. Adapted from [57] with permission from The Royal Society of Chemistry.

In a very recent study, Çakıroğlu et al. constructed a mesoporous TiO_2_ (MTiO_2_) structure with enhanced surface area which improved both GOx and gold NPs immobilization [56]. MoS_2_ was added for visible light harvesting, while gold NPs aimed to improve the photonic efficiency of the PEC system. The multiple heterojunctions of the MTiO_2_-gold NPs-MoS_2_ system enhanced the PEC response towards glucose of the biosensor, which exhibited a detection limit of 1.2 μM and a broad linear range (0.004–1.75 mM). Because MTiO_2_ was synthesized by using tannic acid—a green and cheap material—as a template, their results encourage sustainable strategies for porous material preparation. Another recent work also proposed the use of an advanced TiO_2_-based material for GOx immobilization [57]. More specifically, the authors aimed to provide the biosensor with a higher number of exposed enzyme active sites by means of 3-dimensional (3D) hollow-out titanium dioxide (TiO_2_) nanowire clusters (NWc) on a Ti wire mesh, as illustrated in Figure 3A. The enzymatic reaction could occur with high efficiency on the resulting mesh electrode, which allowed excellent diffusion of glucose and products around the immobilized enzyme. As a result, glucose could be detected with ultrahigh sensitivity in the range between 0 and 2 mM (Figure 3C,D). Moreover, the sensor showed remarkable short- and long-term stability.

### 3.2. Acetylcholine Esterase

Acetylcholine esterase (AChE) is a hydrolase involved in the termination of nerve impulses that catalyzes the hydrolysis of the neurotransmitter acetylcholine to acetate and choline. Its activity is affected by various inhibitors, such as organophosphorus and carbamate compounds used as pesticides and nerve agents (because they lead to the accumulation of acetylcholine, disrupting the neurotransmission). As enzymatic sensors can detect not only the substrate but also the enzyme inhibitors, biosensors based on AChE inhibition can be used for the detection of different analytes by measuring the relative difference between the response in the absence and in the presence of the inhibitor [58,59]. Regarding photoelectrochemical sensors, since Pardo-Yissar et al. demonstrated that AChE could be combined with semiconductor QDs for photoelectrochemical biosensing of an enzyme inhibitor [60], many biosensors using new photoactive hybrid materials have been reported for the detection of organophosphate pesticides (OPs) [20,35,61], aflatoxin B_1_ [62], and for AChE activity studies linked to the investigation of neurodegenerative diseases [63,64,65].

**Figure 4:**
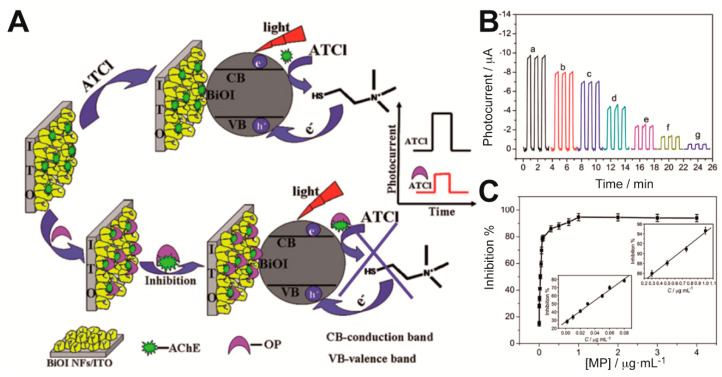
(**A**) Schematic of the photobioelectrochemical (PEC) biosensing principle using Acetyl Choline Esterase (AChE)-bismuth oxyiodide BiOI nanoflake arrays (BiOINFs)/indium-doped tin oxide (ITO) photoelectrodes. (**B**) Photocurrent response over time for increasing concentrations of methyl parathion. (**C**) Inhibition as a function of methyl parathion concentration and linear calibration plots. Adapted with permission from ref. [20].

Gong et al. integrated AChE within a nanostructured porous network of crossed bismuth oxyiodide BiOI nanoflake arrays (BiOINFs) in the design of a highly sensitive biosensor for the detection of an organophosphate pesticide [20]. A 3D network of BiOINFs turned out as an excellent matrix for the enzyme immobilization, which enhanced mass transport and AChE loading on the photoelectrode. Moreover, BiOI exhibits good visible light harvesting properties. As depicted in Figure 4A, the photocurrent of AChE-BiOINFs/ITO electrodes increased in presence of the enzyme substrate acetylthiocholine (ATCl) as a consequence of the hole scavenging properties of the product of the enzymatic reaction (thiocholine) upon irradiation; when an organophosphate pesticide (methyl parathion) was added, an irreversible inhibition effect impaired the enzymatic production of the hole scavenger and that was reflected as a decrease in the photocurrent. The relative difference between the photocurrent values in the absence and presence of the inhibitor methyl parathion (MP) was proportional to its concentration in the ranges 0.001–0.08 μg mL^−1^ and 0.3–1.0 μg mL^−1^ (Figure 4B,C). A detection limit of 0.04 ng mL^−1^ was reported. On the same enzyme inhibition strategy, the use of CdSe@ZnS QDs and graphene nanocomposites [61] and laser-induced TiO_2_-decorated graphene (LITG) [28] was also proposed for the determination of OPs. The simple and scalable preparation method of the latter photoelectrode by direct-laser-writing of LITG on ITO, in which graphene greatly improved the photoresponse of the semiconductor (detection limit of chlorpyrifos: 5.4 pg mL^−1^), could be very promising for PEC assays, although the immobilization of the enzyme was not considered.

A highly sensitive and selective self-powered PEC biosensor for OPs based on an enzymatic fuel cell was also reported [35]. They used (PEDOT)-sensitized CdS QDs forming a bilayer heterojunction—which promoted electron-hole separation and prevented charge recombination—as the photoanode and AChE immobilization platform. For the biocathode, they employed multiwalled carbon nanotubes, gold nanoparticles and bilirubin oxidase (BOx). The electrons generated in the photoanode by the enzyme product thiocholine could be transferred to the biocathode, where O_2_ was reduced by BOx, and as a consequence a high open circuit voltage (E^OCV^) was produced. The variation in the E^OCV^ in the presence of different concentrations of the inhibitor chlorpyrifos was used for the determination of the OP. Their PEC biosensor showed a wide linear range (0.00005 to 0.1 μg mL^−1^) and a detection limit of 0.012 ng mL^−1^.

In a different approach, Zhao et al. proposed a PEC enzymatic sensor aiming at preserving the optimal activity of the enzyme in the absence of the inhibitor by the use of antibodies [66]. AChE antibodies (anti-AChE) were immobilized, instead of the enzyme, on the surface of a photoelectrode consisting of a BiOI nanoflakes/TiO_2_ nanoparticles p-n heterojunction. The presence of the inhibitor methyl parathion in a sample solution containing AChE and acetylthiocholine, in which the photoelectrode was immersed for the immunoreaction with anti-AChE, led to a decrease in the photocurrent that allowed the inhibitor determination (limit of detection: 0.015 ng mL^−1^). This strategy could be extended to the study of the enzymatic activity or inhibition of other enzymes on condition that antibodies are appropriately immobilized.

The inhibitory effect of aflatoxin B_1_ on AChE activity was also exploited for its detection, although in a great lesser extent than for OPs sensing. One PEC biosensor based on TiO_2_ nanotubes, gold nanoparticles and AChE immobilized by crosslinking with glutaraldehyde was reported for the determination of this toxin (Yuan coatings 2018). AFB_1_ competitively inhibited the enzyme and could be determined in the range 1–6 nM with a detection limit of 0.33 nM. The performance of their biosensor competes well with more costly methods for AFB_1_ detection.

Furthermore, the study of AChE inhibition may be crucial in neurodegenerative disease research, because the dysfunction of this enzyme disturbs the cholinergic neurotransmission (i.e., involving neurotransmitter acetylcholine), which is related to the pathogenesis of neurodegenerative disorders such as Parkinson’s disease (PD). A few PEC biosensors have been proposed as simple and sensitive platforms for the study of AChE activity in the presence of neurotoxins or Cd^2+^ ions. As an example, a hybrid photoelectrode for the evaluation of AChE inhibition by two endogenous neurotoxins ((R)-Sal and (R)-NMSal)—which have been believed to play a role in PD—was constructed by using nitrogen and fluorine co-doped TiO_2_ nanotubes (TNs), Ag nanoparticles and AChE [63]. By measuring the photocurrent variations observed as a result of AChE inhibition and the subsequent decrease of thiocholine concentration (the product of acetylthiocholine hydrolysis which acts as an electron donor to scavenge the holes in the valence band of TNs), (R)-Sal and (R)-NMSal could be determined with a detection limit of 0.1 nM and 0.2 nM, respectively. Their results showed that the inhibition by these endogenous neurotoxins was reversible and mixed (competitive-uncompetitive). The inhibition constants were also calculated (K_i_ = 0.35 μM for (R)-Sal and 0.12 μM for (R)-NMSal). The same group reported the preparation of a nanomaterial composed of TNs modified with ZnO nanorods for the immobilization of AChE and the investigation of the effect of Cd^2+^ ions on its activity [64]. The results obtained with their PEC system revealed that Cd^2+^ had an activation effect on AChE activity at low concentration, whereas it had an inhibitory effect at high concentration.

### 3.3. Protein Kinases

Kinases are enzymes that catalyze the transfer of phosphate groups from ATP to other biomolecules such as amino acids in substrate peptides or proteins, sugars, nucleotides or lipids; the case of protein kinases (PKs) have been matter of photoelectrochemical biosensors. Abnormalities in protein kinase activity and the phosphorylation process are related to many diseases, including cancer, diabetes and Alzheimer’s disease [67]. With the aim of determining kinase activities in a simple, rapid and sensitive way and of screening its inhibitors, various photochemical biosensors have been proposed.

Most PEC biosensors for PK activity employed graphite-like carbon nitride (g-C_3_N_4_) as photoactive material [29,68,69], occasionally combined with TiO_2_ to facilitate the effective charge separation and for recognition of the phosphorylated peptide after the PK-catalyzed reaction [70,71]. Yin et al. developed a visible-light activated PEC biosensor based on g-C_3_N_4_, the specific recognition molecule Phos-tag-biotin and avidin modified alkaline phosphatase (streptavidin-ALP) for signal amplification [29]. They constructed a g-C_3_N_4_-AuNPs-ITO electrode whereby a substrate peptide could bind (via the AuNPs and -SH groups of the peptide residues) and then, protein kinase A (PKA) transferred one phosphate group from ATP to the peptide. Subsequently, Phos-tag-biotin identified the phosphate group and the streptavidin-ALP was further captured on the electrode surface through the highly specific interaction between avidin and biotin. Finally, the immobilized ALP catalyzed the conversion of L-ascorbic acid-2-phosphate trisodium salt (AAP) into ascorbic acid (AA), which acted as electron donor and provided one electron to capture the photo-generated hole of g-C_3_N_4_, resulting in an increase of the photocurrent. PKA was thus selectively and sensitively detected (the detection limit was 0.015 U mL^−1^) through the relationship between the photocurrent and PKA concentration. In another work from the same group, the interaction between phosphorylated g-C_3_N_4_ (P-g-C_3_N_4_) nanoparticles and a PKA-induced phosphorylated peptide (P-peptide) triggered by Zr^4+^ ion coordination [68] was proposed as a simple method for PKA activity biosensing. However, the detection limit reported could be improved (0.077 U mL^−1^) and the preparation of P-g-C_3_N_4_ was time-consuming. Moreover, they developed a PEC biosensor that used a g-C_3_N_4_-TiO_2_ composite, as both photoactive material and P-peptide conjugation platform, and a signal amplification strategy triggered by a polyamidoamine (PAMAM) dendrimer and ALP (which catalyzes the production of AA, an electron donor for the generation of the photoelectrochemical response) [70]. The PKA-catalyzed phosphorylation was performed in solution, instead of on the electrode surface, to simplify the experimental procedure and improve the contact between the reactants. Nevertheless, the separation process carried out before the capture of the P-peptide on the electrode (by the use of magnetic beads and carboxypeptidase Y for the hydrolysis and release of the P-peptide) was tedious and actually made the detection process more complicated.

**Figure 5:**
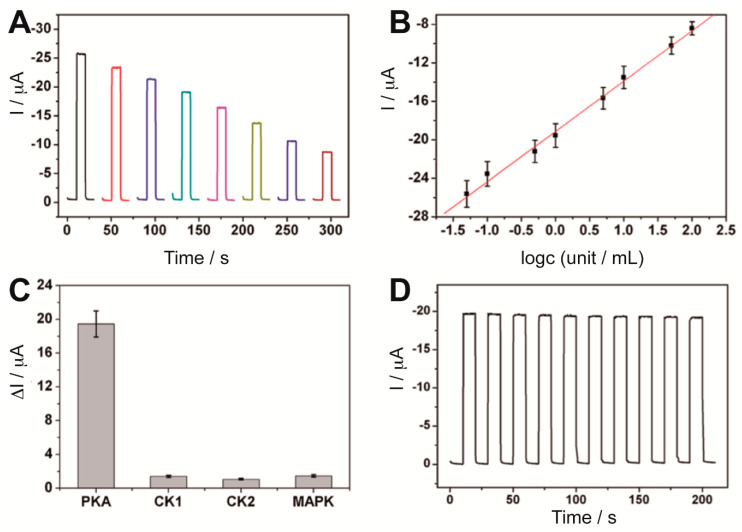
(**A**) Photocurrent response of the PEC biosensor based on biotinylated Phos-tag specific recognition for protein kinase A (PKA)-phosphorylated peptides in the presence of increasing concentrations of PKA (from left to right). (**B**) Photocurrent vs. logarithm of PKA concentration calibration plot. (**C**) Biosensor selectivity after incubation of the peptide-Bi_2_S_3_-AuNPs-ITO electrodes with different protein kinases. (**D**) Stability of the photocurrent response upon chopped-irradiation. Reprinted with permission from ref. [72].

The specific biotin-streptavidin interaction proposed in ref. [29] had been also used by Zhou et al. [72] in a simple label-free PEC biosensing method. Kinase-induced phosphopeptides, previously immobilized on a Bi_2_S_3_-AuNPs-ITO electrode, could bind to a biotinylated Phos-tag in the presence of Zn^2+^ and then streptavidin could be captured on the electrode surface, resulting in a decrease in the photocurrent due to the blocking of streptavidin towards the electron donor AA diffused to the Bi_2_S_3_ surface. The response was related to the phosphorylation extent and therefore to the PKA activity (Figure 5A,B), which could be detected with a detection limit of 0.017 U mL^−1^. Furthermore, the sensor showed good selectivity when tested with other protein kinases and acceptable stability (Figure 5C,D).

Yan et al. further improved the sensitivity achieved by PK sensors and developed a highly sensitive PEC biosensor for PKA activity detection based on Au NPs localized surface plasmon resonance (LSPR) enhancement and dye sensitization [67]. They constructed a TiO_2_-ITO electrode for the immobilization of the peptide and subsequent phosphorylation catalyzed by PKA. Then DNA was conjugated onto AuNPs and specifically coordinated to the P-peptides on the electrode via Zr^4+^ ions. [Ru(bipy)_3_]^2+^ was intercalated into the DNA grooves and harvested visible light to produce excited electrons that injected into TiO_2_ conduction band, resulting in a strong photocurrent. The LSPR and fast electron transfer kinetics provided by AuNPs further improved the photocurrent efficiency and amplified the response. Their biosensor showed extremely low background signals and a detection limit of 0.005 U mL^−1^.

Metal-organic frameworks (MOFs), a class of organic-inorganic hybrid crystalline porous materials, were also used to improve the sensitivity of PK PEC biosensors. For instance, Zr-based metal-organic frameworks (UiO-66) containing [Ru(bipy)_3_]^2+^ in the pores were selected in the design of a biosensor based on surface defect recognition and multiple signal amplification [73]. The surface defects on the ZrO clusters in UiO-66 enabled the binding of the phosphate groups of the peptide previously immobilized on a TiO_2_-ITO electrode. Moreover, the high surface and porosity of the UiO-66 enhanced the amount of [Ru(bipy)_3_]^2+^, which injected excited electrons into the TiO_2_ semiconductor; therefore, that increased the photocurrent and the sensitivity of the biosensor, which presented a detection limit as low as 0.0049 U mL^−1^ and a linear range from 0.005 to 0.0625 U mL^−1^. As a final example, Wang et al. employed gold nanoparticle-decorated zeolitic imidazolate frameworks (Au-ZIF-8) for the immobilization of the substrate peptide on a ITO electrode modified with carbon microspheres [71]. Then, a g-C_3_N_4_-TiO_2_ nanocomposite specifically interacted with the PKA phosphorylated peptide and provided a strong PEC response under visible light. The sensitivity of their biosensor was poorer than that of the UiO-66-based system previously described (0.02·U mL^−1^) but the detection range was significantly wider (0.05 and 50 U mL^−1^).

The PEC biosensors introduced here also performed the detection of PKA activities in cell lysates, which is promising for drug discovery applications, disease diagnosis and evaluation of therapeutic efficiency. Furthermore, these detection and inhibition screening methodologies can be extended to other kinases by changing the substrate peptide.

### 3.4. Lactate Detection

Lactate monitoring is of great importance in medical diagnosis and sports medicine. For instance, lactate is an indicator of traumatic brain injury [74,75] and its levels inform on the training status of athletes. State-of-the art lactate biosensors are moving towards non-invasive point-of-care (POC) detection and wearable systems. To improve the selectivity, both lactate oxidase (LOx) or lactate dehydrogenase (LDH) enzymes are used [76]. The scarce number of PEC enzymatic sensors for lactate detection that have been developed over the last decade uses the latter enzyme. However, wearable POC systems based on LDH and photoelectrochemical principles have not been reported yet for non-invasive lactate monitoring.

The first PEC LDH biosensor that demonstrated its practical applicability in real samples used a TiO_2_ nanoparticle-multiwall carbon nanotube composite as immobilization matrix for LDH [77]. The system showed that the LDH co-substrate nicotinamide adenine dinucleotide (NAD^+^) can be regenerated from the NADH produced during the biocatalytic reaction at a moderate potential (0.2 V vs. Ag/AgCl) by the photoexcited holes of the composite. The biosensor exhibited good long-term stability, high selectivity, a dynamic range of 0.5–120 μM, a sensitivity of 0.0242 μA μM^−1^, and a detection limit of 0.1 μM.

**Figure 6:**
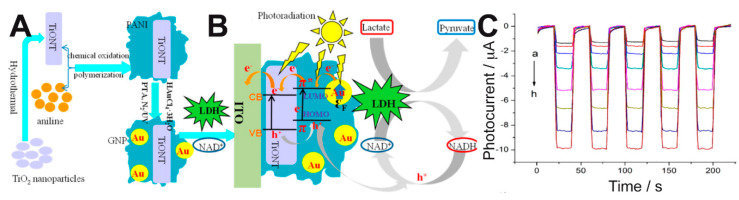
(**A**) Schematic of the preparation of TiO_2_ nanotubes (TiONT)-polyaniline (PANI)-gold nanoparticles (GNPs) composites. (**B**) SPR-enhanced lactate biosensing mechanism of the TiONT-PANI-GNPs/lactate dehydrogenase (LDH)/nicotinamide adenine dinucleotide (NAD^+^)/ITO system comprising PEC cosubstrate regeneration. (**C**) Photocurrent response of the PEC biosensor in the presence of increasing concentrations of lactate. Adapted with permission from [78]. Copyright (2016) American Chemical Society.

Zhu et al. immobilized LDH, NAD^+^ and a ternary composite onto ITO electrodes to develop a PEC biosensor that showed enhanced performance compared to other electrochemical biosensors for lactate [78]. The composite consisted of TiO_2_ nanotubes (TiONTs), gold nanoparticles (GNPs)—which provided a surface plasmon resonance effect (SPR)—and polyaniline (PANI) with excellent electrochromic properties (Figure 6A). This system allowed the efficient regeneration of NAD^+^ and the amplification of the photocurrent response, as depicted in Figure 6B, and it responded to a broad range of lactate concentrations (Figure 6C). The linear range, sensitivity, and detection limit of their method were 0.5–210 μM, 0.0401 μA μM^−1^, and 0.15 μM, respectively.

Furthermore, lactate dehydrogenase can use redox proteins, such as cytochrome c (cyt c), as electron acceptors. A platform that coupled cyt c and pyridine-functionalized CdS nanoparticles was combined with cytochrome-dependent lactate dehydrogenase and allowed the detection of millimolar concentrations of lactate [79]. The system generated oxidation photocurrents (at 0 V vs. SCE and λ = 420 nm) that were enhanced in the presence of increasing concentrations of lactate, as a result of the enzymatic regeneration of reduced cyt c.

### 3.5. Photo-Biosensors for Specific Applications 

Besides the examples shown for the most common enzymatic sensors, there have been some other enzymatic systems developed for photoelectrochemical biosensors. A branch of sensors has focused on monitoring enzymes and their activity, in addition to protein kinases and AChE, already mentioned earlier. The very early optical fiber-based example was designed to detect the presence of catalase [17]. The optical fiber was modified by a partial chemical etching, allowing an interstitial space for the solution containing a sensitizer. The external layer of gold was exposed to a solution with tris(2,2′-bipyridine)ruthenium, afterwards the optical fiber was irradiated with an Ar laser and the internal silica layer provided the photons to excite the electrons of Ru(bpy)_3_^2+^, which reduced O_2_ present in the solution to H_2_O_2_. The gold ring was then used to detect the in-situ generated H_2_O_2_. The sensor was able to quantify catalase activity by measuring the decrease of H_2_O_2_ concentration. Other photoelectrochemical systems have been designed for detecting protease activity, specifically tyrosinase and thrombin, which selectively cleaves arginine-glycine amide bonds [23]. The photoelectrode comprised ITO glasses modified with multiple layers of the sequence CdS and mercaptopropionic acid. After the final CdS layer was deposited a peptide ended in 4-phenyl was immobilized on the surface to serve as protease sensor. Tyrosinase oxidizes the radical to an ortho-quinone derivative, and thrombin cleaves the peptide chain. Both modifications impact the photoelectrochemical response. Detection of tyrosinase limited at 1.5 μg·mL^−1^ and yielded a linear range from 2.6 to 32 μg·mL^−1^; whereas thrombin was detected at 1.9 μg·mL^−1^ and gave a linear range from 4.5 to 100 μg·mL^−1^. Another example where photo-biosensors are used to detect enzymes relied on ITO electrodes as foundation for NiWO_4_ nanostructures, which comprised the photoelectrodes [30] and showed a suitable ability to oxidize uric acid, which served as sacrificial electron donor and allowed the photocurrent. An immunosensor specific for neuron-specific enolase (NSE) was placed on the top of the nanostructures. Upon the presence of the NSE the surface of the electrode gets blocked by the immunoreceptors activity and the photocurrent is hindered. This structure yielded a sensitivity of 0.12 ng mL^−1^. Another enzyme worth monitoring is a cancer marker like type IV collagenase, which is related to liver, breast, colon, lung carcinomas, and leukemia [80]. To do so, an ITO electrode was layer by layer modified with CdTe QDs and a synthetic peptide containing the specific sequence Gly-Pro-Ala, which is the cleavage target of collagenase. To one end of the sequence arginine amino acids were added to be modified with silver nanoparticles. The detection took place by a photoelectrochemical current increase caused by the cleavage of the peptide, which released the silver nanoparticles and allowed a larger radiation to reach the CdTe-ITO electrodes. The photocurrent increase was coherent with the variation in the impedance spectra of the surface. The photosensor yielded a limit of detection of 96 ng mL^−1^ and a linear detection range from 0.5 to 50 μg mL^−1^.

Enzymes are not the only biomolecules that have been a target for photo-biosensors; proteins, peptides and key oligomers have also been matter of research due to its huge relevance in diagnosis. A first example is carcinogenic biomarkers, which have also been addressed with this technology. An ITO electrode modified with a layer of graphene oxide and a second layer of chitosan-Cd^2+^ has been used against a carcinoembryonic antigen (CEA), which served as recognition molecule [21]. The target molecule was the antibody for CEA. When the target molecule is not present, an artificial antibody loaded with horseradish peroxidase (HRP) linked to the surface. The authors promoted this way the reduction of sodium sulfite with H_2_O_2_ catalyzed by HRP, yielding sodium sulfide, which formed QDs with the existing cadmium in the chitosan layer. The appearance of CdS allowed the photooxidation of ascorbic acid, added as revealing agent. A GOx-based photo-biosensor has been used to detect the cancer biomarker α-fetoprotein (AFP) [81]. AFP is a glycoprotein which excess flags a high probability of hepatic carcinoma or endodermic sinus tumor. The sensor consisted in TiO_2_ coupled with an AFP-CdTe-GOx conjugate that includes labels antibodies for AFT and GOx attached to CdTe QDs so its signal can be amplified. The electrode was coated with a layer of chitosan to provide a biocompatible matrix suitable for AFP antibody binding. CdTe QDs improve visible light absorption, thus avoiding the irradiation of the electrode with UV light, which is harmful against enzymes. In addition, quick electron transfer grants enhanced charge separation due to matching energy levels between CdTe and TiO_2_, improving photocurrent response. Furthermore, in the presence of glucose, GOx catalyzes the production of H_2_O_2_ that acts as an electron donor and scavenges the photogenerated electron holes in CdTe QDs valence band, which enhances the photocurrent response even more. The electrodes tested turned out to be long-lasting, highly reproducible and with good sensitivity.

Nucleic acids’ related activity has also been a matter for photo-biosensors. ITO electrodes modified with Bi_2_S_3_ and antibodies specific for methylated DNA have been used to detect the enzyme DNA methyltransferase [22], as the malfunction of this enzyme is related to several diseases and cancer development. The activity of DNA methyltransferase was detected by treating a DNA palindrome single stranded probe, which was methylated by the enzyme and later on linked to a biotinylated complementary sequence. The double-stranded modified sequence was trapped on the photoactive surface loaded with antibodies, and the exposed biotin was used to link alkaline phosphatase. The addition of the revealing probe, phosphorylated ascorbic acid, allowed for the photodetection of the resulting ascorbic acid, which enhanced the photoelectrochemical current if present. Malfunction of the DNA methyltransferase enzyme yielded no immunorecognition, no alkaline phosphatase, and no photooxidation of ascorbic acid. The revealing system was used for peptide detection also on different photoactive materials [25]. In this case an ITO electrode modified with macroporous TiO_2_ loaded with complex quantum dots CdSeTe@CdS@ZnS was used. The photoelectrode was loaded with polyethyleneimine (PEI) and later with a biotinylated peptide for leukemia recognition. The signal was transduced with an equivalent alkaline phosphatase and ascorbic acid oxidation system. In another example, a photo-biosensor designed for hydroxymethylated DNA was presented by using ITO electrodes, which were modified with WS_2_ and gold nanoparticles [27]. On the top of the surface, a DNA probe was immobilized to match the methylated DNA target. This system yielded a linear response in the photocurrent from 0.01 to 100 nM concentrations. Further on, the DNA-modified electrode could also be used to detect glycosyl transferase activity, since this enzyme can use the hydroxymethyl derivative of the DNA and substitute it by a sugar derivative, which can be detected with boronic acid-terminated quantum dots. 

Finally, some other biosensors are devoted to detection of small biomolecules that usually act as biomarkers for several conditions and their monitoring can maintain or improve our health. A formaldehyde biosensor based on formaldehyde dehydrogenase was proven by replacing the NAD cofactor by a CdS-covered gold electrode [18], although this work focused on optimizing the enzyme-CdS interface and its light-dependence rather than developing the analytical conditions of the biosensor. Nitrite is an important analyte to monitor in environmental and food chemistry. An example of nitrite detection used a cytochrome C as recognition molecule deposited on a nanosponge modified with graphene-TiO_2_ nanowires. Upon illumination, the detection limit of nitrite was 0.225 mM and a linear range from 0.5 μM to 9 mM was achieved. Uric acid is a biomolecule that works as biomarker of purine metabolism, and when it is out of range can anticipate gout or other cardiovascular condition [24]. The sensor was built on an ITO electrode coated with several kinds of ZnS nanostructures and the enzyme uricase, which oxidizes the uric acid to allantoin, CO_2_ and H_2_O_2_. The best performing electrode was modified with ZnS urchin-like nanostructures, which upon illumination showed a limit of determination of 45 nM and a linear range from 0.01 to 0.54 mM. Another sensor [32] was prepared using semiconducting CuO nanotubes by oxidation of copper foil in two steps: a wet etching and further annealing. The work was focused on photoelectrode development using the enzyme xanthine oxidase as model reaction for guanine detection.

Lactose determination in dairy products and in particular in those called “lactose-free” is drawing more and more attention because of lactose intolerance problems. Very recently Çakıroğlu et al. have investigated an effective PEC strategy for lactose detection for the first time [82]. They developed a PEC biosensor for glucose and lactose consisting of TiO_2_ modified with gold nanoparticles and a layer of MnO_2_/g-C_3_N_4_ for the co-immobilization of glucose oxidase and β-galactosidase. Lactose measurements could be performed at low potential (−0.4 V vs. Ag/AgCl) with good sensitivity (detection limit of 0.23 μM) and linear range (0.008–2.50 mM).

**Figure 7:**
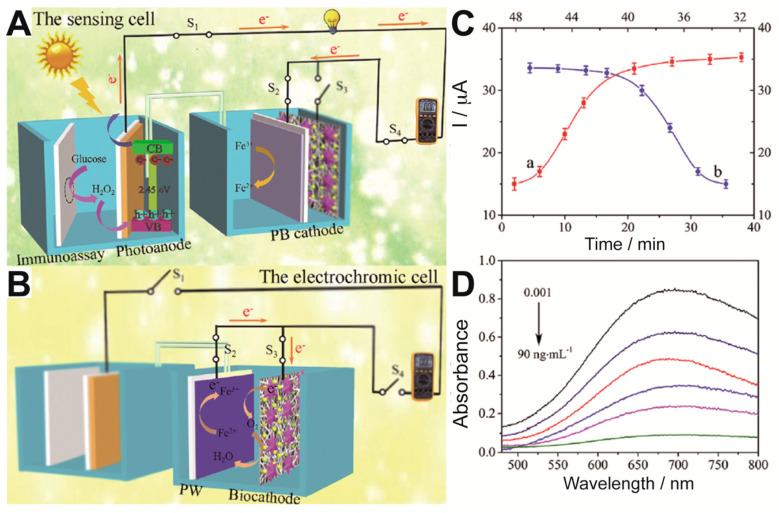
(**A**) Analytical principle of the photoelectrochemical sensing cell triggered by an immunoreaction and connected to a digital multimeter readout. (**B**) Analytical principle of the readout electrochromic cell. (**C**) Time optimization of (a) glucose oxidation and (b) immunosensing processes. (**D**) Electrochromic sensor decreasing absorbance of Prussian Blue, reduced to Prussian White. Adapted from ref. [36].

N-methylglycine, also known as sarcosine, is a natural amino acid present in many organisms and plays a role in some metabolic paths like glycine synthesis or degradation. In addition, it may serve as biomarker of prostate cancer [26]. A photoactive biosensor built on ITO electrodes covered subsequently with layers of NiO, CuInS_2_ and sarcosine oxidase was tested. It should be noted that the interface NiO-CuInS_2_ is a p-p type heterojunction. The system worked by reducing the photocurrent due to the enzymatic activity of sarcosine oxidase, which competed with the photoelectrode for O_2_ and depleted the substrate for the photoelectrode. The photoelectrode offered a limit of detection of 0.008 mM and a linear range from 0.01 to 1 mM. Typical interferences were tested successfully, which can be attributed to the selectivity of sarcosine oxidase. Hydrogen peroxide is also a very interesting molecule for detection, as it is a byproduct of oxidases, substrate of peroxidases and intermediate of many enzymatic cascades. Li el al. [33] prepared arrays of TiO_2_ nanotubes by anodic oxidation of Ti foil and temperature crystallization for 2 h at 450 °C in aerobic conditions. The nanotubes were coated with polydopamine-HRP mix by incubation in solutions of dopamine and later on HRP. The HRP activity oxidized the dopamine, yielding an insoluble product that decreased the photocurrent. The sensor offered a 0.7 nM detection limit and a linear range from 1 nM to 50 μM. This is not the only example of photo-biosensors using TiO_2_ nanotubes synthesized from titanium foil; another example has covered it with chitosan and cross-linked HRP to it with glutaraldehyde, by a double Schiff-base imine formation [34]. In this case, the sensor works by feeding HRP with H_2_O_2_, sodium sulfite and cadmium ytride, which produces CdS that precipitates and increases the photocurrent. The presence of the herbicide asulam avoids the formation of sulfide from sulfite, decreasing the photocurrent. Finally, a very recent work devoted to carcinoembryonic antigen demonstrates a dual electrochromic biosensor where a photoelectrochemical cell plays a role for both powering and sensing the device [36]. The photoanode comprised Ni:FeOOH/BiVO_4_ nanocomposites and was set to power a cathode made of Prussian Blue (Figure 7). The detection of the carcinoembryonic antigen was performed with specific antibodies loaded with glucose oxidase set to form a sandwich structure when the antigen appears. Then glucose was fed into the anodic chamber. If the secondary antibody is linked, which happens only when the antigen has been trapped, H_2_O_2_ is produced by the glucose oxidase enzymatic reaction. H_2_O_2_ then acts as electron donor and is oxidized in the photoelectrode, increasing the current upon illumination. The sensor needed for dual optimization in time incubation, setting that it needs 45 min for immunologic process and 35 min for glucose oxidation by GOx (Figure 7C). The photocurrent generated fed the cathode, where the Prussian Blue was reduced to Prussian White, making the blue color disappear (Figure 7D).

## 4. Conclusions

Photoelectrochemical biosensors have shown a very recent development, which came later than other biosensors due to their higher complexity and difficulty to achieve. Nevertheless, they provide better performance with respect to background signal suppression and the ability to switch the sensor ON and OFF. Moreover, the larger number of components opens the door to tailor on-demand sensors that are not only better suited to specific applications than regular biosensors, but have also shown new ways to create and analyze a sensing signal. Future work will focus on new interfaces electrode-semiconductor and semiconductor-macrobiomolecule—where the optimization can be carried out—while increasing the sensitivity and selectivity of the sensor. Another research trend that allows the inclusion of light-dependent sensors is the selection of the excitation wavelength, which opens plenty of possibilities of multi-sensing platforms by a mere decomposition of white light.

## Figures and Tables

**Table 1:** Performance of various glucose photo-biosensors.

Electrode	Limit of Detection	Linear Range	Ref.
Pt/ZnO/GOx	5.6 μM	N.A.	[37]
Pt/CdS/GOx	1 μM	1 μM–2.5 mM	[38]
ITO/CdSe@CdS/GOx	0.05 mM	1–8 nM	[39]
FTO/CdTe QD/GOx	0.04 mM	0.1–11 mM	[40]
ITO/ZnS/GOx	0.02 mM	0.1–5.5 mM	[41]
FTO/TiO_2_ NW/GOx	0.9 nM	N.A.	[42]
FTO/ZnO IOPC/GOx	N.A.	0.4–4.5 mM	[43]
GCE/p-HT/GDH	1.5 μM	5 μM–1 mM	[44]
GCE/ZnS-CdS QD/GDH	4.0 μM	0.010–2.0 mM	[45]
PGE/ZnS-CdS QD/GDH	0.05 mM	0.2–8.0 mM	[46]
ITO/PbS QD/GOx	0.3 μM	1 μM–10 mM	[47]
ITO/g-C_3_N_4_/GOx	0.01 mM	0.05–15 mM	[48]
ITO/Co_3_O_4_/CNT/GOx	0.20 μM	0–4 mM	[49]
FTO/⍺-Fe_2_O_3_/NB/GDH	25.2 μM	0.25–2.0 mM	[50]
FTO/R/A TiO_2_/GOx	0.019 mM	1–20 mM	[51]
ITO/NDC-TiO_2_ NPs/GOx	13 nM	0.05–10 μM	[52]
GCE/CoP-PCN/GOx	1.1 μM	0.05–0.7 mM	[53]
GCE/g-C_3_N_4_/ZnIn_2_S_4_/GOx/HRP	0.28 μM	1 μM–10 mM	[54]
FTO/BiVO_4_/GOx	0.73 μM	1–400 μM	[55]
ITO/Au NP/MoS_2_/GOx	1.2 μM	4 μM–1.75 mM	[56]
3D hollow-out TiO_2_ NWc/GOx	8.7 μM	0–2 mM	[57]

N.A: No data available.
